# Development and validation of a Multiplex Real-Time PCR for HTLV-1/2 confirmatory diagnosis

**DOI:** 10.1186/1742-4690-11-S1-P105

**Published:** 2014-01-07

**Authors:** Maurício Cristiano Rocha Júnior, Rodrigo Haddad, Virginia Mara de Deus Wagatsuma, Oswaldo Massaiti Takayanagui, Svetoslav Nanev Slavov, Katia Kaori Otaguiri, Evandra Strazza Rodrigues, Dimas Tadeu Covas, Simone Kashima

**Affiliations:** 1 Hemocentro de Ribeirão Preto, Universidade de São Paulo (USP), Ribeirão Preto, São Paulo, Brazil; 2 Faculdade de Ciências Farmacêuticas de Ribeirão Preto - Universidade de São Paulo (USP) Ribeirão Preto, São Paulo, Brazil; 3 Universidade de Brasília, Faculdade de Ceilândia – UNB/FCE, Brasília, Distrito Federal, Brazil; 4 Faculdade de Medicina de Ribeirão Preto - Universidade de São Paulo (USP) Ribeirão Preto, São Paulo, Brazil

## 

Accurate diagnostic tests are powerful tool to control the spread of the human T-lymphotropic virus (HTLV) infection. In Brazil, the currently applied diagnostic algorithm for HTLV-1/2 screening is based on serological tests (enzyme-linked immunosorbent assay followed by Western Blot). However this algorithm is unsuitable due to its high cost and the elevated rate of Western Blot (WB) indeterminate results. Nevertheless, molecular techniques such as real-time PCR (qPCR) can overlap these drawbacks because of their high sensitivity and specificity. Several studies have described different qPCR protocols for HTLV-1/2 diagnosis but they lack suitable validation. For this reason, we developed and validated a qualitative multiplex qPCR platform for simultaneous detection and discrimination of the HTLV-1/2 infection in a single reaction tube. A total of 73 HTLV-positive samples and 100 samples obtained from non-infected individuals were tested. The detection limit was one copy/reaction for HTLV-1 and 10 copies/reaction for HTLV-2. We observed a high and similar efficiency between the single and multiplex format as well as the cycle treshold and *r^2^* values. In addition, this molecular platform reached 100% of sensitivity and specificity. In conclusion, the developed method using one tube multiplex qPCR (confirmatory and discriminatory) for HTLV-1/2 was validated. It showed low cost and high sensitivity and specificity compared to previously described assays and to the traditional confirmatory method (WB). Therefore, this platform can be a supportive tool for the current confirmatory methods adopted.

